# Expression levels of matrix metalloproteinase-9 in human gastric carcinoma

**DOI:** 10.3892/ol.2014.2768

**Published:** 2014-12-04

**Authors:** SU-ZUAN CHEN, HUAI-QI YAO, SEN-ZHI ZHU, QIU-YUAN LI, GUANG-HUA GUO, JING YU

**Affiliations:** Department of Gastroenterology, The First Affiliated Hospital, Shantou University Medical College, Shantou, Guangdong 515041, P.R. China

**Keywords:** gastric carcinoma, matrix metalloproteinases-9, semi-quantitative polymerase chain reaction, immunohistochemistry, malignancy

## Abstract

The present report investigated the correlation between the expression levels of matrix metalloproteinase (MMP)-9 in gastric carcinoma patients and the clinicopathological characteristics. Forty-five samples of gastric carcinoma and distal gastric mucosa tissue, and 10 samples of healthy gastric mucosa tissue were analyzed using semi-quantitative polymerase chain reaction, as well as immunohistochemical and hematoxylin and eosin staining. MMP-9 protein levels in serum samples from the same patients were quantified by enzyme-linked immunosorbent assay. The present report identified that MMP-9 expression was markedly higher in the gastric carcinoma tissue (86.67%) than in the adjacent healthy tissue (10.00%). A positive association was identified between the level of MMP-9 protein expression and the depth of cancer invasion (P<0.05). Furthermore, the preoperative serum levels of the MMP-9 protein in the gastric carcinoma tissue were correlated with the tumor-node-metastasis stage and occurrence of lymph node metastasis (P<0.01). Data from the present report indicates that MMP-9 may be key in gastric carcinoma malignancy, and implies that MMP-9 may serve as a novel biomarker in the diagnosis and prognosis of gastric carcinoma.

## Introduction

Gastric carcinoma is one of the most common malignancies worldwide. Although the early diagnosis and treatment of gastric cancer has improved in recent years, it remains one of the leading causes of cancer-related mortality in countries, such as China and Japan (1). The aggressive behavior of cancer cells is predominantly attributed to their capacity to invade and metastasize. Metastasis is a complex process that involves the spread of a cancer to a site distant from its origin. Reduced cell-cell adhesion and degradation of the extracellular matrix (ECM) are essential mechanisms for the occurrence of cell invasion and metastasis, leading to the dissemination of a cancer via the blood vessels or the lymph nodes (2).

Matrix metalloproteinase (MMP)-9 is the largest member of the MMPs, an extensive family of zinc-dependent proteolytic enzymes. Previous studies have validated that MMP-9 degrades the principal components of the ECM, collagen types IV and V, and gelatin and, thus, is closely associated with tumor cell invasion and metastasis (3–5). Furthermore, it has been reported that MMP-9 serum levels in the peripheral blood can be used for preoperative prognosis (6,7). The MMP-9 expression level is elevated in non-small cell lung cancer and is associated with lymph node metastasis, although, MMP-9 expression levels are not associated with postoperative survival rate (8). MMP-9 is associated with the growth, invasion and metastasis of gastric carcinoma (9), however, there are few reports regarding preoperative serum levels of MMP-9 in gastric carcinoma. In the current study, the expression of MMP-9 in gastric carcinoma samples was analyzed and the correlation between preoperative serum levels of the MMP-9 protein and the clinicopathological characteristics of gastric carcinoma was investigated.

## Patients and methods

### Patients and sample collection

Forty-five primary gastric carcinoma patients, who had undergone a gastrectomy at The First Affiliated Hospital of Shantou University Medical College (Shantou, China) between June 2005 and January 2006, were involved in the present study. Thirty cases were male and 15 cases were female, with a mean age of 60.4 years. Fourteen patients had a family history of cancer. None of the patients had received chemotherapy or radiotherapy prior to the surgery and no additional malignancies were evident. All patients were informed of the nature of the study and provided written informed consent, and the study was approved by the ethics commitee of the Medical College of Shantou University.

Peripheral venous blood (3 ml) was collected from each patient prior to the surgery and stored at −75°C for the enzyme-linked immunosorbent assay (ELISA; Sino Biological Inc., Bejing, China). Tumor tissue samples were obtained immediately from the resected specimens and distal healthy gastric mucosa tissue was collected from a distance of >5 cm from the tumor. For immunohistochemistry, the tissue samples were fixed with 10% formalin, dehydrated with gradient alcohol, cleared with xylene and embedded in paraffin. In addition, 10 specimens of peripheral serum and gastric mucosa tissue from 10 healthy individuals were randomly selected to serve as controls. Classification of the tumors was conducted according to the tumor-node-metastasis (TNM) staging system issued by the International Union Against Cancer in 1997 (10).

### Semi-quantitative reverse transcription-polymerase chain reaction (RT-PCR) analysis of MMP-9 expression

Total RNA was extracted using TRI Reagent^®^ (Molecular Research Center, Inc., Cincinnati, OH, USA) followed by complementary DNA synthesis using the Moloney murine leukemia virus reverse transcriptase (Thermo Fisher Scientific, Pittsburgh, PA, USA). PCR was performed using Taq polymerase (Takara Biotechnology Co., Ltd., Dalian, China) under the following conditions: 94°C for 2 min; 22 cycles (for β-actin) or 32 cycles (for MMP-9) of 94°C for 30 sec, 57°C for 30 sec and 72°C for 40 sec; followed by 72°C for 5 min. β-actin served as an internal control. The PCR product lengths for MMP-9 and β-actin were 462 and 592 bp, respectively and were sequenced by Invitrogen Biotechnology Co., Ltd. (Shanghai, China). The primers for the PCR were as follows: Sense, 5′-CACCCTTGTGCTCTTCCCTG-3′ and anti-sense, 5′-GGATACCCGTCTCCGTGCT-3′ for MMP-9; sense, 5′-CCAAGGCCAACCGCGAGAAGATGAC-3′ and anti-sense, 5′-AGGGTACATGGTGGTGCCGCCAGAC-3′ for β-actin. Each PCR product was examined by agarose gel electrophoresis and images were captured using a digital camera (PowerShot A2400; Canon Inc., Tokyo, Japan). The protein band intensities were analyzed using Image J software (National Institutes of Health, Bethesda, MD, USA).

### Immunohistochemical staining of MMP-9

Serial sections were cut from the paraffin-embedded tissues at a thickness of 5 μm, and routine hematoxylin and eosin (H&E) staining was conducted prior to immunohistochemical staining. The staining procedure was performed according to the immunohistochemical EliVision™ two-step method (11) and a tissue section with a known MMP-9 expression level served as the positive control. Staining with phosphate-buffered saline, as a substitute for the primary antibody, served as the negative control. Cells exhibiting a brown-stained cytoplasm, at a higher intensity than the non-specific background staining, were regarded as positively stained and tumors exhibiting positive staining in >10% of the total number of tumor cells were regarded as positive for MMP-9 expression. The human monoclonal anti-MMP-9 and poly-horseradish peroxidase secondary antibodies were purchased from Maxim Biotechnology Development Co., Ltd. (Fuzhou, China).

### ELISA of MMP-9

The concentration of the MMP-9 protein in the peripheral serum was measured using a double-antibody sandwich ELISA method (Wuhan Boster Biological Technology, Ltd, Wuhan, China) according to the manufacturer’s instructions. Following coloration with 3,3′-diaminobenzidine, the optical density value was measured at a wavelength of 450 nm and a standard curve was determined to calculate the concentration of MMP-9 protein.

### Statistical analysis

Statistical analysis was performed using SPSS software (version 10.0; SPSS, Inc., Chicago, IL, USA). Student’s t-test was applied for data analysis between the two groups, χ^2^ and Fisher’s exact tests were applied for numeration data, and a Pearson correlation coefficient was used to analyze the correlation between gene expression and the protein level. P<0.05 was considered to indicate a statistically significant difference.

## Results

### MMP-9 expression levels in tissue samples

**Figure 1:**
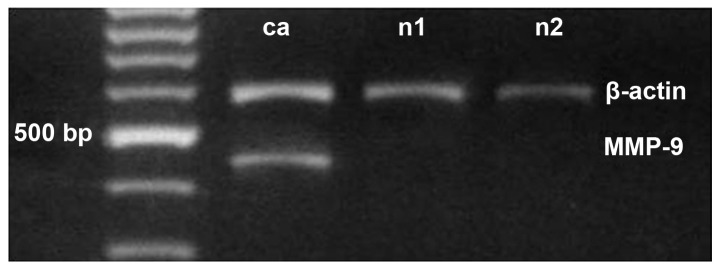
mRNA expression of MMP-9 and β-actin extracted from gastric carcinoma tissue (ca), distal gastric mucosa tissue (n1) and healthy gastric tissue (n2). MMP-9, matrix metalloproteinase-9.

Semi-quantitative RT-PCR (Fig. 1) identified MMP-9 expression in 48.9% (22/45) of the gastric carcinoma tissues, 13.3% (6/45) of the distal gastric mucosa tissues (>5 cm from the gastric tumor) and 10% (1/10) of the healthy gastric mucosa control tissues. The mean level of MMP-9 mRNA in the gastric carcinoma tissues (0.42±0.65) was significantly higher than in the distal (0.22±0.76; P<0.05) and control tissues (0.03±0.06; P<0.05; Table I).

**Figure 2:**
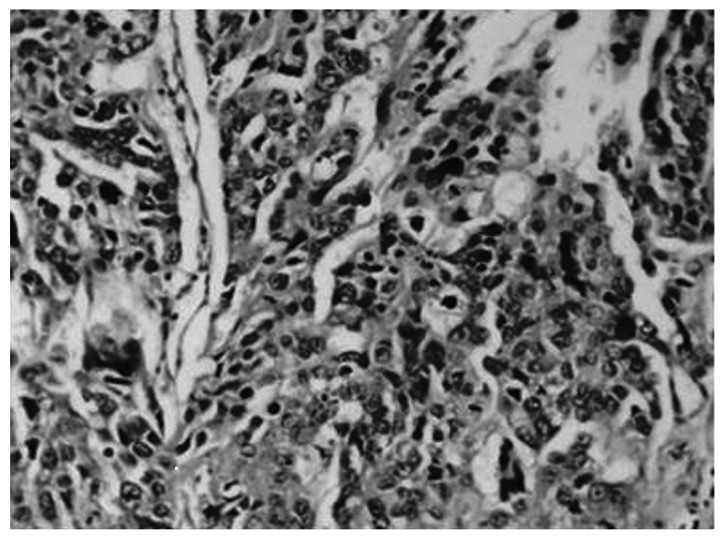
Hematoxylin and eosin staining of matrix metalloproteinase-9 in gastric carcinoma tissue, as shown by light gray grains in the cytoplasm (magnification, ×200).

**Figure 3:**
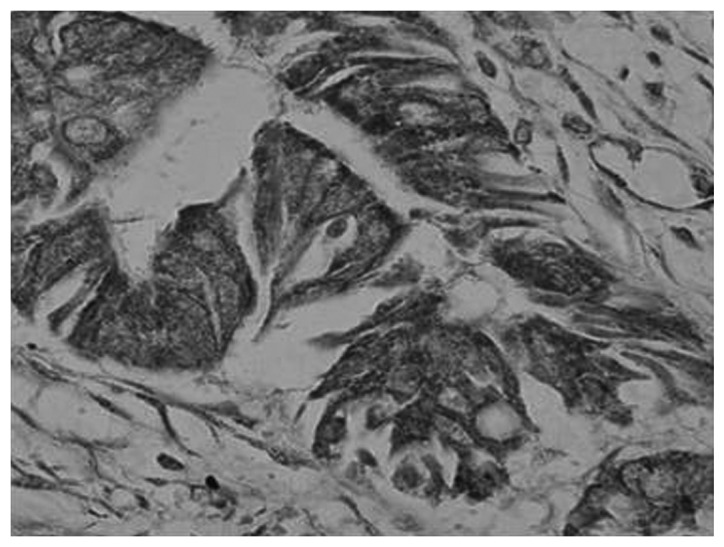
Immunohistochemical staining of matrix metalloproteinase-9 in gastric carcinoma tissue, as shown by dark grains in the cytoplasm (magnification, ×400).

H&E (Fig. 2) and immunohistochemical (Fig. 3) staining for MMP-9 was observed in the majority of gastric carcinoma tissues. The expression rate of MMP-9 was 86.67% (39/45) in the gastric carcinoma tissues, 26.67% (12/45) in the distal gastric mucosa tissues and 10% (1/10) in the healthy gastric mucosa tissues (Table I). Correlation between MMP-9 expression levels and the clinicopathological characteristics of the tumor revealed that patients with seromembranous invasion had a significantly higher proportion of MMP-9-positive tumor cells (92.31%; 36/39) than patients without seromembranous invasion (50.00%; 3/6) (P<0.05). However, no significant difference in the levels of MMP-9 expression was identified with respect to the tumor size, family history of tumors, TNM stage, or occurrence of lymph node or distant metastasis (Table II).

### Correlation between preoperative serum MMP-9 protein levels and clinicopathological features of gastric carcinoma

The mean preoperative serum MMP-9 protein levels in gastric carcinoma patients (0.41±0.26 μg/ml) was significantly higher than those of the control group (0.15±0.04 μg/ml; P<0.05) (Table I). In addition, the serum concentration of MMP-9 protein in patients with an advanced TNM stage (511.42±251.52), and lymph node metastasis (498.55±265.77) was significantly higher than that in the patients at an early TNM stage (305.22±223.99) and exhibiting no lymph node metastasis (300.30±205.62), respectively (P<0.01). However, there was no significant correlation between the serum MMP-9 protein level and other clinicopathological characteristics, such as the tumor size, a family history of tumors, localization of the tumor, tumor differentiation, degree of invasion or distal metastasis (Table II).

### Correlation between MMP-9 expression levels in gastric carcinoma tissues and preoperative serum MMP-9 protein levels

The expression level of MMP-9 in gastric carcinoma tissues and the preoperative serum was higher when compared with the healthy tissues. However, the correlation between MMP-9 mRNA levels in gastric carcinoma tissues and the preoperative serum MMP-9 levels was not identified to be statistically significant (r=0.13; P=0.394).

## Discussion

Degradation of the ECM and basement membranes is a critical step in tumor invasion and metastasis. The process of metastasis depends on the activity of various proteolytic enzymes, particularly serine and cysteine proteases, and MMP. MMPs, a family of closely associated enzymes that degrade the ECM, are considered to be important in facilitating tumor invasion and metastasis, and are associated with tumor invasion, lymph node metastasis and survival of gastric carcinoma (12). Previously, studies have identified a significant correlation between the expression of MMP-2, -7 and -9, and the depth of tumor invasion, vessel invasion, lymph node and distant metastasis, TNM stage and microvessel density, indicating that these markers may be significant in cell migration in gastric carcinoma (13–15). Zymographic analysis of the expression levels of MMP-9 and active MMP-2 demonstrated a proportional increase with tumor grade and invasiveness in bladder cancer (16). MMP-9 mRNA expression levels in lymphocytes tended to be higher in malignant pleural effusions of lung cancer when compared with healthy pleural effusions (17). Consistent with the findings of the above-mentioned reports, the present study demonstrated that MMP-9 was highly expressed in gastric carcinoma tissues and preoperative serum when compared with distal and healthy tissue.

Correlation between the MMP-9 expression levels and the clinicopathological parameters of tumors did not identify any significant difference, with the exception of the depth of tumor invasion. The incidence of MMP-9 positive expression was observed to be greater in tumors with seromembranous invasion compared with non-invasive tumors, indicating that MMP-9 may contribute to invasion and tissue infiltration in gastric carcinoma. Furthermore, the present study identified that patients with gastric carcinoma exhibited higher preoperative peripheral serum MMP-9 levels compared with the control groups, and demonstrated that the serum MMP-9 levels correlated with the TNM stage and occurrence of lymph node metastasis. These data indicate that serum protein expression levels may contribute to the malignant tumor phenotype, as reported in previous studies (15,18). MMP-9 mRNA and serum expression were upregulated in gastric carcinoma, however, the present study did not observe a correlation between MMP-9 expression levels in the gastric carcinoma tissues and those in the preoperative serum. This may be due to mechanisms, in addition to the influence of cancerous tissue on serum MMP-9 protein secretion (19), such as the action of macrophages, leucocytes and other inflammatory cells (20,21). In the present study, it was identified that MMP-9 expression tended to be higher in patients with a positive family history of cancer compared with patients without, however, no significant correlation was observed. Additionally, the present study demonstrated that MMP-9 expression levels in distal tissues were significantly higher than those of the healthy control group, indicating that abnormal gene activation may be involved, possibly in a similar manner to MMP-9 overexpression in benign gastric lesions (22).

Previous investigations, regarding how differences between plasma and serum samples influence the diagnostic and prognostic performance of MMP-9, identified that plasma MMP-9 levels were elevated in gastric cancer patients when compared with control subjects, whilst serum MMP-9 levels did not differ between the groups (23–25). By contrast, in non-small cell lung cancer, serum MMP-9 levels were demonstrated to be higher in patients with metastatic cancer (26,27), indicating that detection of the preoperative plasma MMP-9 expression level may serve as an improved marker for tumor progression when compared with serum MMP-9 levels.

In conclusion, the present study demonstrated that MMP-9 protein levels in the preoperative serum and mRNA expression in carcinoma tissue were higher than the healthy controls, and were correlated with specific clinicopathological features of gastric carcinoma. The data indicates that MMP-9 has potential as a diagnostic marker and therapeutic molecular target for management of gastric carcinoma.

## Figures and Tables

**Table I:** Matrix metalloproteinase-9 parameters of the gastric carcinoma, distal tissue and control groups (mean ± standard deviation).

Group	Cases, n	mRNA value	Positive expression, n (%)	Protein concentration, μg/ml
Gastric carcinoma	45	0.42±0.65^a^	39 (86.67)^a^	0.41±0.26^a^
Distal tissue	45	0.22±0.76	12 (26.67)^b^	
Control	10	0.03±0.06	1 (10.00)^b^	0.15±0.04

a P<0.01 vs. the control group;

b P<0.05 vs. the distal tissue and control group.

**Table II:** Correlation between the MMP-9 protein levels in the peripheral serum and clinicopathological features (mean ± standard deviation).

Feature	Cases, n	Mean gray value	Positive rate of MMP-9 protein, % (n)	Serum (μg/ml)
Tumor size, cm				
<5	22	0.47±0.61	81.82 (18/22)	378.14±278.61
≥5	23	0.75±0.59	91.30 (21/23)	432.71±238.05
Family history of tumor				
Negative	31	0.55±0.56	83.87 (26/31)	391.81±283.28
Positive	14	0.75±0.72	92.86 (13/14)	437.52±192.69
Affected region				
Upper part	21	0.48±0.44	85.71 (18/21)	450.39±289.75
Middle-lower part	24	0.73±0.72	87.5 (21/24)	367.22±223.97
Differentiation				
Well and moderate	27	0.60±0.62	81.48 (22/27)	380.22±257.50
Poor or none	18	0.79±0.61	94.44 (17/18)	444.75±259.12
Depth of invasion				
No seromembranous invasion	6	0.25±0.28	50.00 (3/6)^b^	303.47±202.06
Seromembranous invasion	39	0.67±0.63	92.31 (36/39)^b^	421.81±250.33
TNM stage				
I + II	23	0.54±0.60	78.26 (18/23)	305.22±223.99^a^
III + IV	22	0.68±0.63	95.45 (21/22)	511.42±251.52^a^
Lymphatic metastasis				
Negative	21	0.58±0.61	85.71 (18/21)	300.30±205.62^a^
Positive	24	0.64±0.62	87.50 (21/24)	498.55±265.77^a^
Distant metastasis				
Absent	43	0.59±0.62	86.05 (37/43)	396.28±254.94
Present	2	1.00±0.11	100.00 (2/2)	615.72±297.53

MMP-9, matrix metalloproteinase-9; TNM, tumor-node-metastasis.

a P<0.01 and

b P<0.05.
